# Fruit and vegetable consumption and serum vitamin A in lactating women: A cross‐sectional survey in urban China

**DOI:** 10.1002/fsn3.2532

**Published:** 2021-08-20

**Authors:** Chenlu Yang, Ai Zhao, Hanglian Lan, Jian Zhang, Zhongxia Ren, Ignatius Man‐Yau Szeto, Peiyu Wang, Yumei Zhang

**Affiliations:** ^1^ Department of Nutrition and Food Hygiene School of Public Health Peking University Beijing China; ^2^ Vanke School of Public Health Tsinghua University Beijing China; ^3^ Inner Mongolia Dairy Technology Research Institute Co., Ltd. Hohhot Inner Mongolia Autonomous Region China; ^4^ Yili Maternal and Infant Nutrition Institute Inner Mongolia Yili Industrial Group Co., Ltd. Hohhot Inner Mongolia Autonomous Region China; ^5^ Department of Social Medicine and Health Education School of Public Health Peking University Beijing China; ^6^ Beijing Key Laboratory of Toxicological Research and Risk Assessment for Food Safety School of Public Health Peking University Beijing China

**Keywords:** fruit, lactating women, serum vitamin A, vegetable, *zuo yuezi*

## Abstract

During the lactation period, healthy eating behavior is essential to maternal and child health. However, Chinese lactating women may have some traditional food restrictions. Our aims were to evaluate the fruit and vegetable consumption of Chinese lactating women and to examine the associations between fruit and vegetable consumption and serum vitamin A concentrations. A total of 885 participants were included. Dietary intakes were assessed during the same time frame as blood collection via a one‐time 24‐h dietary recall (24HDR) and a semiquantitative food frequency questionnaire (SFFQ), respectively. Serum vitamin A concentrations were assessed with high‐performance liquid chromatography. Based on 24HDR, 64.7% and 85.5% of lactating women did not consume the appropriate amount of fruits and vegetables, respectively. New mothers who adopt *zuo yuezi* behavior during the first month were negatively associated with fruit consumption. The median (25th to 75th) dietary vitamin A intake was 349.5 (202.5–591.4) μg RAE/day. Vegetable contributed 24.9% and fruit 4.8% of the dietary vitamin A intake. The median (25th to 75th) serum vitamin A concentration was 1.92 (1.61–2.30) μmol/L. 24HDR assessments of total fruit and vegetable consumption, and fruit consumption were positively associated with higher serum vitamin A concentrations, respectively (β = 0.200, 95%CI = 0.077, 0.323, *p* = .001; β = 0.241, 95%CI = 0.008, 0.474, *p* = .044). These positive associations were replicated in the SFFQ assessments (β = 0.102, 95%CI = 0.016, 0.188, *p* = .020; β = 0.215, 95%CI = 0.088, 0.341, *p* = .001). Chinese lactating women had inappropriate fruit and vegetable consumption. Fruit and vegetable consumption was associated with serum vitamin A concentrations.

## Graphical abstract

New mothers who adopt zuo yuezi behavior during the first month were negatively associated with fruit consumption. The median (25th to 75th) serum vitamin A concentration was 1.92 (1.61–2.30) μmol/L. Fruit and vegetable consumption was associated with serum vitamin A concentrations.

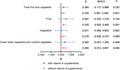

## 1 INTRODUCTION

During the lactation period, healthy eating habits are essential to help mothers rebuild their body stores of nutrients depleted during pregnancy and conserve nutrient stores to ensure breast milk supply without compromising maternal nutrition reserves (Hanson et al., 2015; Yu et al., 2018). In China, lactating women may have some traditional food restrictions, especial for women during *zuo yuezi* or *doing the month*, which is a tradition among lactating women in the puerperium, with a history of more than 2000 years (Tsai & Wang, 2019). Lactating women are encouraged to consume certain foods, such as animal products, various soups, red sugar, and rice wine (Mao et al., 2016; Zhao et al., 2016). These foods have been traditionally regarded as beneficial to breast milk quantity and quality. However, its role still needs to be fully elucidated, and there is a problem with this dietary pattern. For example, fresh fruits or vegetables are not highly recommended because lactating women should avoid “cold” foods. According to traditional Chinese medicine theory, different food properties (such as “warm” and “cold”) can alter the “*Yin‐Yang* balance” in the body (Ding, Niu, et al., 2020; Ding, Indayati, et al., 2020; Liu et al., 2020; Zheng et al., 2017). Childbirth is believed to disturb the “*Yin‐Yang* balance,” and usually, the “cold” foods were related to “*Yin*” (Zheng et al., 2017). For example, the “cold” fruits mainly include banana, blueberry, mulberry, orange, and watermelon, and the “cold” vegetable mainly include cauliflower, celery, Chinese cabbage, cucumber, seaweed, and snow peas. Besides, Some people classify “warm” and “cold” food according to its temperature (Dai, 2019). One research study in China has shown that only one‐third of lactating women consume appropriate amounts of fruits and vegetables (Zhao et al., 2016). In 2018, the Chinese Nutrition Society (CNS) had established the new Chinese Balanced Dietary Pagoda for lactating women (Society 2020). Fruit and vegetable intake should be within 200–400 g per day and 400–500 g per day, respectively, and green leafy vegetables and colored vegetables, such as red and yellow vegetables, should comprise more than 2/3 of the total vegetable intake. However, whether fruit and vegetable intake during lactation meets the new guidelines has not been well‐studied.

Fresh fruits and vegetables are rich in bioactive phytochemicals that may provide many desirable health benefits. One such phytochemical is carotenoid, which is crucial as pro‐vitamin A (Hanson et al., 2015; Tanumihardjo et al., 2016). Vitamin A plays essential roles in normal vision, gene expression, growth and physical development, the maintenance and proliferation of epithelial cells, and immune function, at all stages of life, particularly for lactating women, given both maternal and newborn requirements (Oliveira et al., 2016). A lactating woman's vulnerability to vitamin A deficiency is increased by the transfer of a significant amount of retinol to the infant via breast milk (Ncube et al., 2001). The intake of foods that are rich in pro‐vitamin A (darkly colored vegetables and fruits) is an essential way in which the prevalence of vitamin A deficiency can be curbed (Maina et al., 2019). In industrialized countries, preformed vitamin A accounts for nearly 65% of total vitamin A intake, and carotenoids make up 35% (Weber & Grune, 2012). On the other hand, in developing countries, 70%–90% of vitamin A is consumed as pro‐vitamin A carotenoids (Cabezuelo et al., 2019). One study has shown that the amount of dark green leafy vegetables consumed is the primary determinant of vitamin A intake in women and corresponds to a higher serum retinol status (Stuetz et al., 2019). Therefore, lactating women on a diet with inadequate fruits and vegetables may be prone to inferior serum vitamin A status. However, the association between fruit and vegetable consumption and vitamin A status among Chinese lactating women has not been well‐described.

The relative contribution of pro‐vitamin A to the vitamin A dietary intake depends not only on the amount of fruits and vegetables consumed, but also on the bioavailability and capacity of conversion into retinol of the carotenoids consumed (Olmedilla‐Alonso et al., 2020). For example, one survey among pregnant women in Tanzania reported that there was no significant association between the frequency of vegetable consumption and either plasma retinol or carotenoids; however, the increased consumption of green leafy vegetables with oil, which improves bioavailability, is associated with high plasma retinol levels (Mulokozi et al., 2003). It is therefore necessary to consider animal products and cooking oil when examining the association of fruit and vegetable consumption with serum vitamin A status. Furthermore, some studies only use 24‐h dietary records to calculate dietary intake (Hanson et al., 2016; Mielgo‐Ayuso et al., 2017), which is unlikely to be representative of the usual intake, as the day‐to‐day intake of fruit and vegetable can be highly variable.

Therefore, the objectives of the current study were (1) to evaluate the fruit and vegetable consumption of Chinese lactating women and (2) to examine the associations between fruit and vegetable consumption and serum vitamin A concentrations.

## 2 MATERIALS AND METHODS

### 2.1 Subjects

This study was based on a subset of data of the Young Investigation (YI Study), which was a cross‐sectional survey on health and nutrition status of pregnant women, lactating women, young children aged 0–3 years from 2019 to 2020. Ten cities (Beijing, Chengdu, Guangzhou, Hohhot, Lanzhou, Nanchang, Ningbo, Shenyang, Suzhou, and Xuchang) were selected according to their geographical position and economic level. From the geographical locations, Guangzhou, Suzhou, Ningbo, Nanchang, and Chengdu were located in the South of China, while Shenyang, Beijing, Hohhot, Lanzhou, and Xuchang were located in the North of China. From the economic perspective, Beijing and Guangzhou were the first‐tier cities, Suzhou, Chengdu, and Shenyang were the new first‐tier cities, Ningbo, Lanzhou, and Nanchang were the second‐tier cities, and Hohhot and Xuchang were the third‐tier and fourth‐tier cities, respectively. Within each city, one hospital or one maternal and child healthcare center was selected. The target was to recruit at least 90 lactating women in each city, and lactating women were conveniently recruited according to their visiting time until the number of participants satisfied the sample size. For lactating women, the inclusion criteria were healthy women in the first year postpartum, aged between 20 and 45 years, with singleton delivery, no smoking or alcohol abuse, without mastitis or any infectious diseases, and without cardiovascular or metabolic diseases. We excluded those participants with missing data or extreme outliers for key variables under the purpose of this report. Finally, 885 participants were enrolled in the current study. In our study, sample size calculations were based on a previous work of our research group, and we found 43.2% and 44.6% of lactating women in 3 cities in urban China could not consume the appropriate amount of fruit and vegetable (Zhao et al., 2016). The following formula was used to calculate the sample size (Charan & Biswas, 2013):

$$n=\frac{Z_{1‐\alpha /2}^{2}*p\left(1‐p\right)}{d^{2}}$$

The *α* was set at 0.05 level, and *d* (admissible error) was 0.1*p* here. By taking the proportion of inappropriate fruit intake (43.2%), the total sample size was 506. By taking the proportion of inappropriate vegetable intake (44.6%), the total sample size was 478. The number of lactating women met the calculated sample size.

### 2.2 Data collection

Data were collected from lactating women by trained interviewers using an interviewer‐administered questionnaire. Training of the interviewers, the initial site survey, and preliminary questionnaire testing were completed prior to data collection. Data were double‐entered, and input errors and logic errors during data entry were revised during data screening.

### 2.3 Dietary data

We used a one‐time 24‐h dietary recall (24HDR) to obtain data on food consumption on the day prior to investigation. With the help of trained interviewers, participants were asked to recall all food, beverages, and condiments consumed individually over the previous 24 h using a purposely designed data collection form. The collection table consisted of types of food, beverages, and condiments, time of eating or drinking, ingredients, cooking methods, and quantity of foods, beverages, and condiments consumed. We also used a semiquantitative food frequency questionnaire (SFFQ) to obtain the average daily food consumption in the past month. With the help of trained interviewers, participants were asked to report the frequency (never, or times per day, week, or month) and average intake amounts for each food item in the past month. In the field work, standard‐sized bowls, standard‐sized teaspoons, and illustrated photographs of food items (Ding, Niu, et al., 2020; Ding, Indayati, et al., 2020) were shown to help participants to assess quantities. Food consumption was expressed as grams per day.

In this report, the classification was processed as follows.

1. The fruit group included all fresh fruit products.
2. The vegetable group included green leafy vegetables (broccoli, spinach, chives, watercress, celery leaves, lettuce, water spinach), colored vegetables such as red and yellow ones (pumpkin, carrot, tomato, red pepper), and other groups (cabbage, Chinese cabbage, cauliflower, cucumber, white radish; Yang et al. 2016; Zhang et al. 2010).
3. The total fruit and vegetable group included all fruits and vegetables reported earlier.
4. The animal products group included livestock meat, poultry, fish, shrimp, shellfish, and eggs.
5. The cooking oil group included both animal and vegetable oils.

Dietary vitamin A (μg RAE/day) (RAE stands for retinol activity equivalents) and total energy intake in the past 24 h were collected via 24HDR and calculated on the Chinese Food Composition Table coupled with the nutrition information packaging (Yang, 2009).

### 2.4 Blood sample collection and laboratory analysis

A 5‐mL blood sample under fasting was drawn from the antecubital vein into a vacuum blood collection tube. Blood sample was left standing for 0.5 h before centrifugation at room temperature for 10 min at 956 *g*. Serum was extracted and protected from direct light and stored at −80℃ until analyzed. Serum vitamin A concentrations were assessed with high‐performance liquid chromatography (Waters Acquity UPLC I‐Class TQD) by the internal standard method (retinol‐d3 as the internal standard substance) (Tanumihardjo et al., 2016). Samples (200 μl) were added to EP tubes, 100 μl internal standard was added and then vortexed to mix the contents for 1 min. Then, the mixture was mixed with 1200 μl of n‐hexane solution, vortexed to mix the contents for 10 min, and centrifuged for 10 min at 3,220 *g*. 800 μl of the supernatant were taken to a 96‐well plate, dried under a nitrogen stream and dissolved in 100 μl of acetonitrile. The mixture was vortexed to mix the contents for 1 min, and centrifuged for 5 min at 3,220 *g*. The supernatant of the above samples was taken for LC‐MS/MS analysis. Chromatographic separations were carried out on an ACQUITY UPLC®BEH C18 column, 2.1 × 50 mm, at 40℃. Mobile phase A was 0.1% formic acid aqueous solution and 0.1% formic acid methanol solution, containing 2 mmol/L ammonium acetate. The flow rate was 0.4 ml/min and the injection volume was 2 μl. The laboratory analysis was conducted by a single qualified laboratory (Beijing Health Clinic Laboratory, China).

### 2.5 Other covariates

Essential characteristics of the lactating women were collected, including age, education, family monthly per capita income (Chinese yuan), parity (1, ≥2), and *zuo yuezi* (within the 30 days of birth). Weight and height were measured and used to calculate body mass index (BMI). BMI <18.5, 18.5–23.9, 24–27.9, and ≥28 kg/m^2^ were considered to be underweight, normal weight, overweight, and obese, respectively, according to the Chinese BMI standard. Metabolic equivalent of energy (MET) hours per week were calculated based on the short version of the International Physical Activity Questionnaire (IPAQ; Fan et al., 2014). Besides, participants were asked to report the nutrient supplements they used during lactation. Vitamin A supplements in this study referred to those that contained vitamin A and/or carotenoid. Women who consumed any vitamin A supplements during lactation were considered as users; otherwise, they were regarded as nonusers.

### 2.6 Statistics

SPSS version 26.0 (SPSS Inc., Chicago, IL, USA) was used for analyses. Normality for continuous data was tested before analyses. Values were presented as Mean ± standard deviation (*SD*), Median (25th, 75th), or percentage. Fruit and vegetable consumption based on 24HDR was compared with the recommended daily food intake based on the Chinese Balanced Dietary Pagoda. For single‐factor analyses, the Mann–Whitney U or Kruskal–Wallis H test was conducted to compare the fruit and vegetable consumption of lactating women according to basic characteristics. Chi‐squared analyses were conducted to compare basic characters in lactating women who achieved the recommended amount of fruits and vegetables. Spearman rank correlation coefficients were calculated to reflect the correlation between the 24HDR‐derived and SFFQ‐derived food groups. The Mann–Whitney U or Kruskal–Wallis H test was conducted to compare the serum vitamin A concentration of lactating women by basic characters. Multilevel linear mixed‐effect models were used to assess the association of fruit and vegetable consumption, vitamin A from fruit and vegetable with serum vitamin A concentration. Cities were handled as random effects. Model 0 was not adjusted. Model 1 was adjusted for cooking oil. Model 2 was adjusted for cooking oil and animal products. Model 3 was adjusted for cooking oil, animal products, age, education, family monthly per capita income (Chinese yuan), parity, *zuo yuezi*, BMI, MET hours per week, and vitamin A supplements. Additionally, stratified analyses by vitamin A supplements were performed. The statistically significant difference in this study was set to a *p*‐value <.05.

## 3 RESULTS

### 3.1 Basic characteristics

Table 1 shows the basic characteristics of lactating women. The mean age was 30.3 ± 4.2 years. In general, the level of education was high. The mean BMI was 23.3 ± 3.3 kg/m^2^, with 326 (36.8%) participants categorized as overweight or obese. Besides, around one‐tenth of participants reported the use of vitamin A supplements during lactation.

**TABLE 1:** Basic characteristics of lactating women in this study

Variable	*n*	(%)
Age (years)		
≤30	451	51.0
>30	434	49.0
College or university		
No	219	24.7
Yes	666	75.3
Family monthly per capita income (Chinese yuan)		
<5000	402	45.4
5000–9999	330	37.3
≥10,000	153	17.3
*Zuo yuezi*		
Yes	45	5.1
No	840	94.9
Parity		
1	585	66.1
≥2	300	33.9
BMI		
Underweight or normal weight	559	63.2
Overweight	249	28.1
Obese	77	8.7
Cities		
First‐tier	169	19.1
New first‐tier	270	30.5
Second‐tier	268	30.3
Third‐ or fourth‐tier	178	20.1
Regions		
South	446	50.4
North	439	49.6
Vitamin A supplements		
No	775	87.6
Yes	110	12.4

### 3.2 Fruit and vegetable consumption

Table 2 shows the fruit and vegetable consumption based on 24HDR. Lactating women during *zuo yuezi* had lower consumption of fruits. Lactating women aged ≤30, without college or university education, and with lower family monthly per capita income had lower consumption of vegetables, as well as green leafy vegetables and colored vegetables. Lactating women who resided in the third‐ or fourth‐tier cities had lower consumption of fruits and vegetables, and those who resided in the north of China had lower consumption of fruits and vegetables. Compared with the recommendation of the Chinese Balanced Dietary Pagoda, based on 24HDR, 64.7% and 85.5% of lactating women consumed less than the recommended amounts of fruits and vegetables. As shown in Table 3, lactating women during *zuo yuezi* had a lower proportion of reaching the recommended level of fruits. Those with lower family monthly per capita income had a lower proportion of reaching the recommended level of vegetables. Lactating women who resided in the north of China had lower proportions of reaching the recommended level of vegetables. The correlations of each food group between 24HDR and SFFQ assessments are shown in Supplemental Table 1, and all of the *p*‐values were <0.01.

**TABLE 2:** Fruit and vegetable consumption [g/day, median (25th, 75th)] among lactating women with different characteristics

Variable	Total fruit and vegetables	*p*‐Value	Fruit	*p*‐Value	Vegetables	*p*‐Value	Green leafy vegetables and colored vegetables	*p*‐Value
All	320.0 (188.0, 530.0)		115.0 (0.0, 250.0)		174.0 (90.0, 300.0)		50.0 (5.5, 130.0)	
Age (years)								
≤30	310.0 (155.0, 530.0)	0.032	100.0 (0.0, 265.0)	0.419	155.0 (80.0, 280.0)	<0.001	50.0 (0.0, 115.0)	0.005
>30	336.5 (200.0, 525.0)		120.0 (9.0, 250.0)		195.0 (107.3, 316.3)		62.5 (10.0, 145.0)	
College or university								
No	310.0 (150.0, 475.0)	0.016	100.0 (0.0, 250.0)	0.298	150.0 (70.0, 240.0)	0.001	40.0 (0.0, 100.0)	0.001
Yes	330.0 (186.8, 550.0)		120.0 (0.0, 250.0)		180.0 (100.0, 316.3)		60.0 (10.0, 140.1)	
Family monthly per capita income (Chinese yuan)								
<5000	295.0 (145.0, 500.0)	0.008	100.0 (0.0, 250.0)	0.446	155.0 (80.0, 265.3)	0.002	49.0 (0.0, 110.5)	0.008
5000–9999	346.0 (190.0, 562.5)		140.0 (0.0, 262.5)		180.3 (98.3, 306.3)		60.0 (10.0, 150.0)	
≥ 10,000	350.0 (214.0, 540.0)		120.0 (0.0, 239.0)		205.0 (110.0, 360.0)		75.0 (7.5, 150.0)	
*Zuo yuezi*								
Yes	230.0 (150.0, 435.0)	0.011	70.0 (0.0, 152.5)	0.028	150.0 (95.0, 200.0)	0.171	60.0 (20.0, 102.5)	0.830
No	326.0 (180.0, 534.8)		120.0 (0.0, 250.0)		175.0 (90.0, 300.0)		50.0 (5.0, 130.0)	
Parity								
1	319.0 (174.5, 535.0)	0.610	116.0 (0.0, 250.0)	0.914	165.0 (85.0, 292.5)	0.086	50.0 (5.0, 115.0)	0.053
≥2	323.0 (188.5, 518.8)		107.5 (3.9, 250.0)		194.5 (100.0, 320.0)		60.0 (10.0, 150.0)	
BMI								
Underweight or normal weight	339.0 (180.0, 540.0)	0.106	120.0 (0.0, 260.0)	0.561	180.0 (95.0, 310.0)	0.071	50.0 (8.0, 135.0)	0.714
Overweight	310.0 (180.0, 512.5)		105.0 (0.0, 250.0)		165.0 (86.0, 290.0)		50.0 (3.5, 124.0)	
Obese	268.0 (159.5, 465.0)		100.0 (0.0, 229.0)		158.0 (75.0, 242.5)		40.0 (7.5, 125.0)	
Cities								
First‐tier	312.0 (180.0, 540.0)	0.023	95.0 (0.0, 221.0)	0.202	200.0 (106.5, 330.0)	<0.001	85.0 (19.0, 150.0)	0.002
New first‐tier	330.0 (200.0, 531.0)		140.0 (0.0, 265.0)		180.0 (90.0, 300.0)		58.0 (0.0, 150.0)	
Second‐tier	350.0 (180.0, 568.0)		122.5 (0.0, 277.5)		183.5 (100.0, 327.3)		54.0 (15.0, 129.5)	
Third‐ or fourth‐tier	268.0 (150.0, 540.0)		100.0 (0.0, 221.0)		130.5 (66.5, 330.0)		33.5 (0.0, 150.0)	
Regions								
South	357.5 (215.0, 564.0)	<0.001	130.0 (0.0, 252.3)	0.207	200.0 (110.0, 336.1)	<0.001	72.5 (10.0, 150.8)	<0.001
North	280.0 (150.0, 490.0)		100.0 (0.0, 250.0)		145.0 (75.0, 240.0)		50.0 (5.0, 100.0)	
Vitamin A supplements								
No	310.0 (170.0, 520.0)	0.004	100.0 (0.0, 250.0)	0.004	170.0 (90.0, 295.0)	0.161	50.0 (7.0, 130.0)	0.800
Yes	403.5 (209.8, 574.8)		155.0 (53.8, 298.3)		197.0 (98.3, 350.0)		50.0 (0.8, 121.8)	

Note

Mann–Whitney U or Kruskal–Wallis H test.

**TABLE 3:** Proportion of differences in lactating women who achieved the recommended amount of fruit and vegetables (g/day)

Variable	Fruit consumption			*p*‐Value	Vegetable consumption			*p*‐Value
	<200	200–400	>400		<400	400–500	>500	
Number of participants	573	208	104		757	66	62	
Age (years)								
≤30	50.3	49.5	57.7	0.288	52.0	42.4	46.8	0.190
>30	49.7	50.5	42.3		48.0	57.6	53.2	
College or University								
No	25.5	26.4	17.3	0.181	25.6	19.7	19.4	0.160
Yes	74.5	73.6	82.7		74.4	80.3	80.6	
Family monthly per capita income (Chinese yuan)								
<5000	48.3	38.5	43.3	0.266	47.2	36.4	33.9	0.006
5000–9999	34.2	44.2	40.4		36.6	42.4	40.3	
≥10,000	17.5	17.3	16.3		16.2	21.2	25.8	
*Zuo yuezi*								
Yes	6.6	1.9	2.9	0.014	4.9	9.1	3.2	0.926
No	93.4	98.1	97.1		95.1	90.9	96.8	
Parity								
1	64.9	66.3	72.1	0.184	66.4	57.6	71.0	0.959
≥2	35.1	33.7	27.9		33.6	42.4	29.0	
BMI								
Underweight or normal weight	61.4	64.4	70.2	0.111	62.9	65.2	64.5	0.544
Overweight	29.5	26.9	23.1		28.0	30.3	27.4	
Obese	9.1	8.7	6.7		9.1	4.5	8.1	
Cities								
First‐tier	20.1	19.7	12.5	0.335	18.4	21.2	25.8	0.060
New first‐tier	29.7	30.8	34.6		30.6	30.3	29.0	
Second‐tier	29.7	28.4	37.5		29.5	39.4	30.6	
Third‐ or fourth‐tier	20.6	21.2	15.4		21.5	9.1	14.5	
Regions								
South	49.0	51.0	56.7	0.700	48.0	62.1	67.7	<0.001
North	51.0	49.0	43.3		52.0	37.9	32.3	
Vitamin A supplements								
No	88.8	87.0	81.7	0.125	88.2	81.8	85.5	0.242
Yes	11.2	13.0	18.3		11.8	18.2	14.5	

Note

(1) Based on the Chinese Balanced Dietary Pagoda for lactating women, fruit and vegetable intake should be 200–400 g/day and 400–500 g/day, respectively.

(2) Chi‐squared analyses.

### 3.3 Serum vitamin A concentration

The median (25th to 75th) serum vitamin A concentration was 1.92 (1.61–2.30) μmol/L. As shown in Table 4, compared with lactating women aged younger than or equal to 30, those aged above 30 had a higher serum vitamin A concentration. Increasing BMI was associated with higher serum vitamin A concentration. Meanwhile, lactating women with vitamin A supplements had higher serum vitamin A concentrations than those without vitamin A supplements. Lactating women who resided in the third‐ or fourth‐tier cities had lower serum vitamin A concentrations, and those who resided in the north of China had lower serum vitamin A concentrations.

**TABLE 4:** Serum vitamin A [μmol/L, median (25th, 75th)] among lactating women with different characteristics

Variable	Serum vitamin A	*p*‐Value
Age (years)		
≤30	1.87 (1.58, 2.24)	0.004
>30	1.98 (1.67, 2.35)	
College or university		
No	1.91 (1.56, 2.28)	0.332
Yes	1.93 (1.62, 2.31)	
Family monthly per capita income (Chinese yuan)		
<5000	1.90 (1.59, 2.26)	0.146
5000–9999	1.97 (1.65, 2.33)	
≥10,000	1.94 (1.68, 2.29)	
*Zuo yuezi*		
Yes	2.03 (1.70, 2.37)	0.289
No	1.91 (1.61, 2.30)	
Parity		
1	1.91 (1.60, 2.29)	0.302
≥ 2	1.95 (1.64, 2.32)	
BMI		
Underweight or normal weight	1.90 (1.59, 2.23)	0.002
Overweight	1.96 (1.65, 2.34)	
Obese	2.16 (1.73, 2.61)	
Cities		
First‐tier	1.99 (1.68, 2.29)	<0.001
New first‐tier	2.02 (1.69, 2.44)	
Second‐tier	1.92 (1.66, 2.29)	
Third‐ or fourth‐tier	1.73 (1.44, 2.29)	
Regions		
South	1.98 (1.71, 2.35)	<0.001
North	1.88 (1.55, 2.22)	
Vitamin A supplements		
No	1.90 (1.60, 2.28)	<0.001
Yes	2.10 (1.79, 2.45)	

Note

(1) Mann–Whitney U or Kruskal–Wallis H test.

**FIGURE 1:**
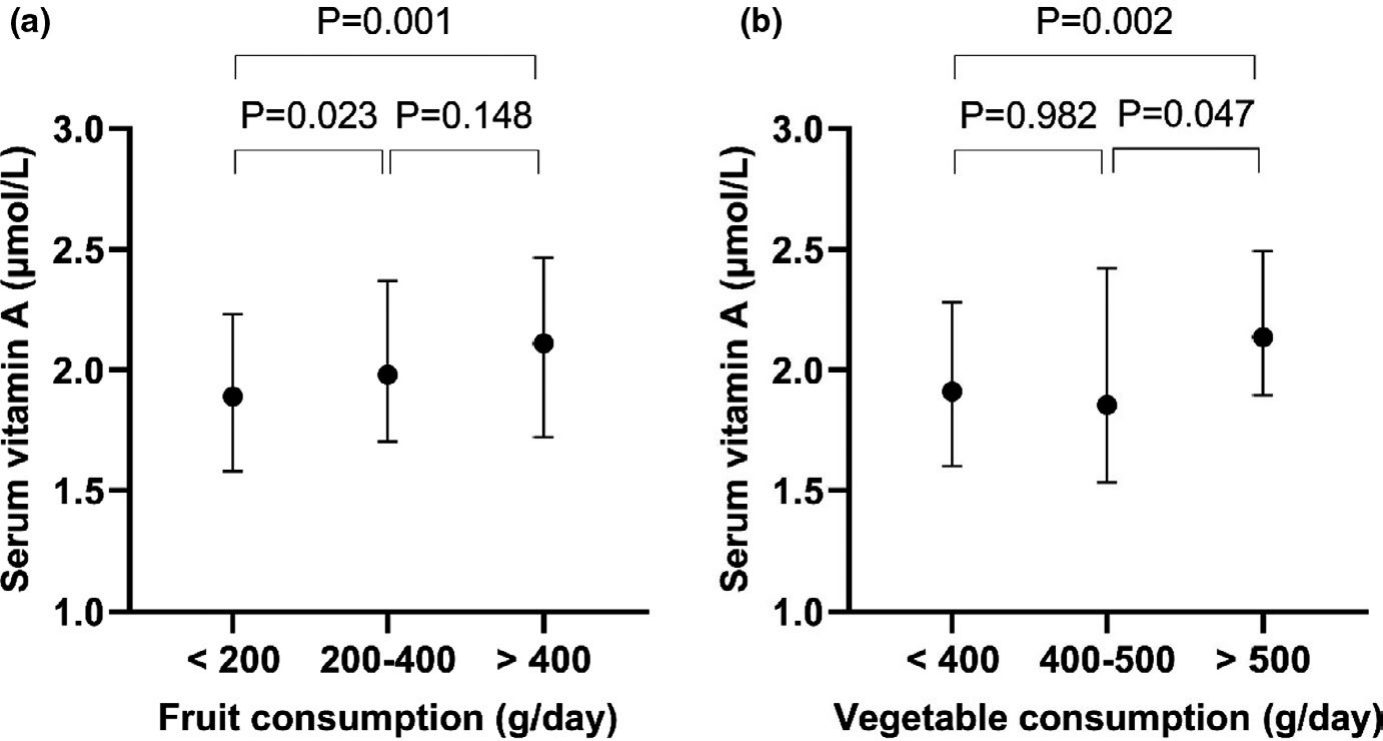
Serum vitamin A [Median (25th, 75th)] in lactating women who achieved the recommended amount of fruit and vegetable. *Note*. (1) Based on the Chinese Balanced Dietary Pagoda for lactating women, fruit and vegetable intake should be within 200–400 g/day and 400–500 g/day, respectively. (2) Mann–Whitney U test

As shown in Figure 1a, the lactating women whose fruit consumption was 200–400 g/day and >400 g/day had higher serum vitamin A concentrations, respectively, compared with those who had a fruit consumption of <200 g/day. As shown in Figure 1b, the lactating women whose vegetable consumption was >500 g/day had higher serum vitamin A concentrations compared with those who had a vegetable consumption of <400 g/day and 400–500 g/day.

### 3.4 Fruit and vegetable consumption in the past 24 hr and serum vitamin A concentration

**FIGURE 2:**
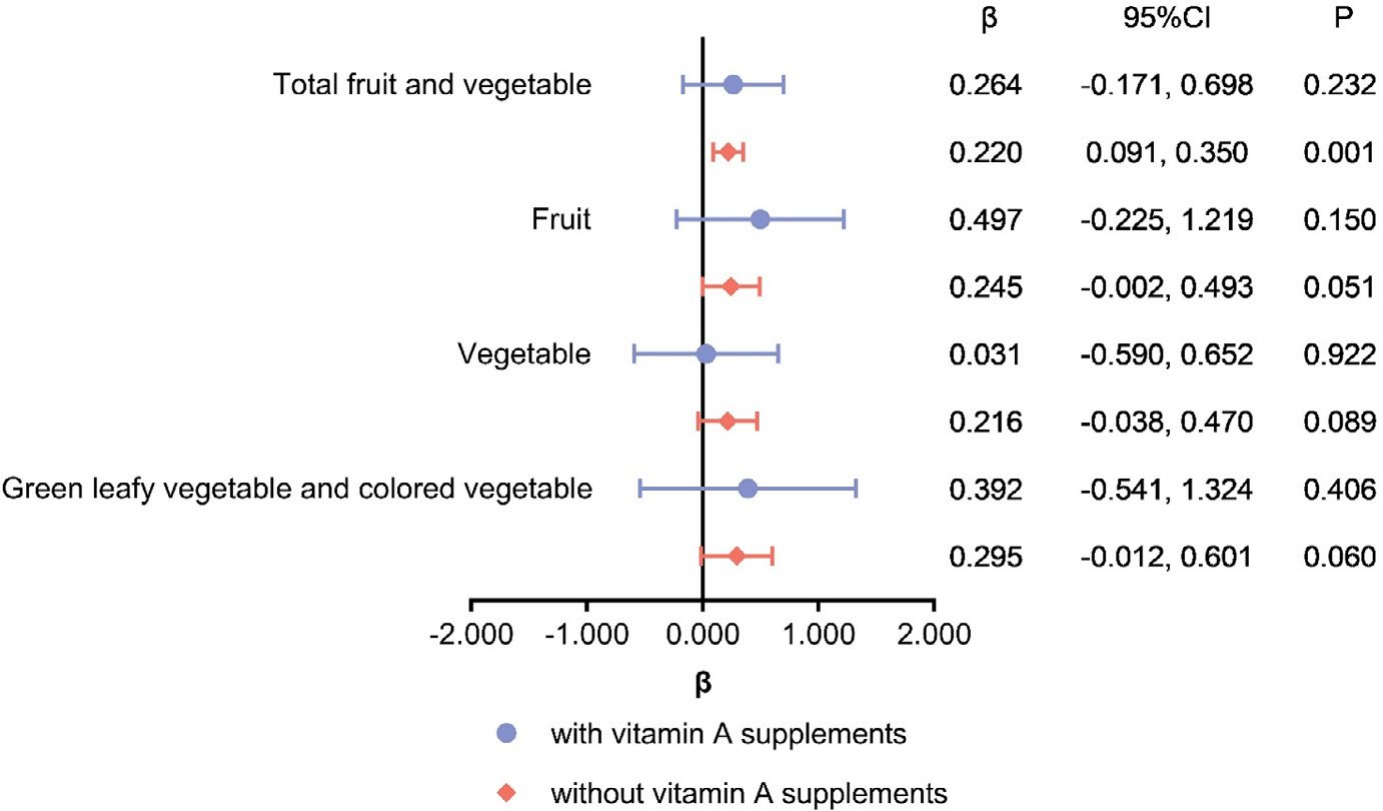
Stratified analysis of association between fruit and vegetable consumption (g/day) based on 24HDR and serum vitamin A concentration (μmol/ml)

Based on 24HDR, fruit, vegetable, animal products, and cooking oil consumption were calculated. After the adjustment of cooking oil, animal products, energy and other possible influential factors at different levels for Models 0–3 (Model 0: no adjustment), higher consumption of total fruits and vegetables, and fruit consumption were associated with higher serum vitamin A concentrations, respectively (Table 5). For vegetable, and green leafy vegetable and colored vegetable consumption, in Models 0, 1 and 2, higher consumption was associated with higher serum vitamin A concentrations; however, this association disappeared in Model 3. The results of the stratified analysis are presented in Figure 2. For women without vitamin A supplements, total fruit and vegetable consumption was positively associated with higher serum vitamin A concentrations.

**TABLE 5:** Association between fruit and vegetable consumption based on 24HDR and serum vitamin A concentration (μmol/mL)

	β	SE	*p*‐Value	95% CI
Total fruit and vegetable consumption (g/day)				
Model 0	0.255	0.060	<0.001	0.137, 0.373
Model 1	0.255	0.060	<0.001	0.137, 0.373
Model 2	0.234	0.060	<0.001	0.116, 0.353
Model 3	0.200	0.063	0.001	0.077, 0.323
Fruit consumption (g/day)				
Model 0	0.291	0.104	0.024	0.051, 0.532
Model 1	0.291	0.104	0.023	0.051, 0.532
Model 2	0.273	0.104	0.031	0.033, 0.513
Model 3	0.241	0.101	0.044	0.008, 0.474
Vegetable consumption (g/day)				
Model 0	0.275	0.106	0.025	0.042, 0.508
Model 1	0.275	0.107	0.025	0.042, 0.508
Model 2	0.241	0.105	0.043	0.009, 0.472
Model 3	0.165	0.113	0.171	−0.082, 0.411
Green leafy vegetable and colored vegetable consumption (g/day)				
Model 0	0.448	0.148	0.003	0.158, 0.738
Model 1	0.447	0.148	0.003	0.157, 0.738
Model 2	0.382	0.149	0.011	0.089, 0.674
Model 3	0.290	0.148	0.050	0.000, 0.581

Note

Model 0, no adjustment.

Model 1, adjusted for cooking oil based on 24HDR.

Model 2, adjusted for cooking oil based on 24HDR and animal products based on 24HDR.

Model 3, adjusted for cooking oil based on 24HDR, animal products based on 24HDR, energy based on 24HDR, age, education, family monthly per capita income (Chinese yuan), *zuo yuezi*, parity, BMI, met‐hours per week, and vitamin A supplements.

### 3.5 Fruit and vegetable consumption in the past month and serum vitamin A concentration

**FIGURE 3:**
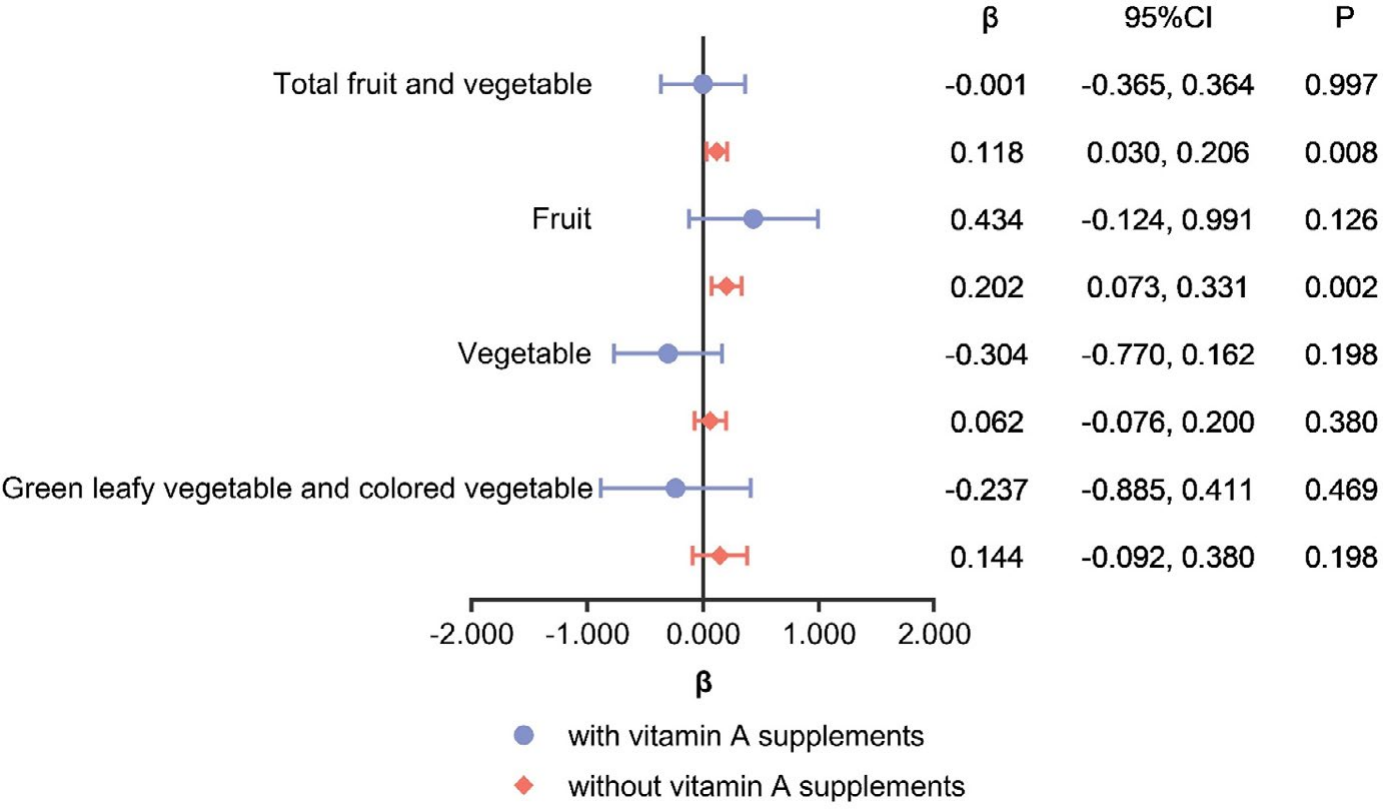
Stratified analysis of association between fruit and vegetable consumption (g/day) based on SFFQ and serum vitamin A concentration (μmol/ml)

Based on SFFQ, the average daily consumption of fruit, vegetable, animal products, and cooking oil in the past month was calculated. As shown in Table 6, after adjustment for possible influential factors, higher consumption of total fruits and vegetables and fruit consumption were associated with higher serum vitamin A concentrations. The results of the stratified analysis are presented in Figure 3. For women without vitamin A supplements, total fruit and vegetable, and fruit consumption were positively associated with higher serum vitamin A concentrations, respectively.

**TABLE 6:** Association between daily consumption of fruit and vegetables based on SFFQ and serum vitamin A concentration (μmol/mL)

	β	SE	*p*‐Value	95% CI
Total fruit and vegetable consumption (g/day)				
Model 0	0.122	0.043	0.005	0.038, 0.207
Model 1	0.125	0.043	0.004	0.041, 0.210
Model 2	0.114	0.044	0.010	0.028, 0.201
Model 3	0.102	0.044	0.020	0.016, 0.188
Fruit consumption (g/day)				
Model 0	0.220	0.065	0.001	0.092, 0.348
Model 1	0.221	0.065	0.001	0.093, 0.349
Model 2	0.212	0.065	0.001	0.084, 0.341
Model 3	0.215	0.064	0.001	0.088, 0.341
Vegetable consumption (g/day)				
Model 0	0.076	0.075	0.357	−0.113, 0.264
Model 1	0.078	0.073	0.336	−0.110, 0.265
Model 2	0.050	0.073	0.524	−0.137, 0.237
Model 3	0.010	0.069	0.893	−0.165, 0.184
Green leafy vegetable and colored vegetable consumption (g/day)				
Model 0	0.178	0.109	0.145	−0.077, 0.433
Model 1	0.179	0.108	0.138	−0.073, 0.432
Model 2	0.149	0.107	0.206	−0.102, 0.400
Model 3	0.094	0.103	0.385	−0.140, 0.329

Note

Model 0, no adjustment.

Model 1, adjusted for daily consumption of cooking oil based on SFFQ.

Model 2, adjusted for daily consumption of cooking oil based on SFFQ and animal products based on SFFQ.

Model 3, adjusted for daily consumption of cooking oil based on SFFQ, animal products based on SFFQ, age, education, family monthly per capita income (Chinese yuan), *zuo yuezi*, BMI, met‐hours per week, and vitamin A supplements.

### 3.6 Dietary vitamin A intake in the past 24 h and serum vitamin A concentration

**FIGURE 4:**
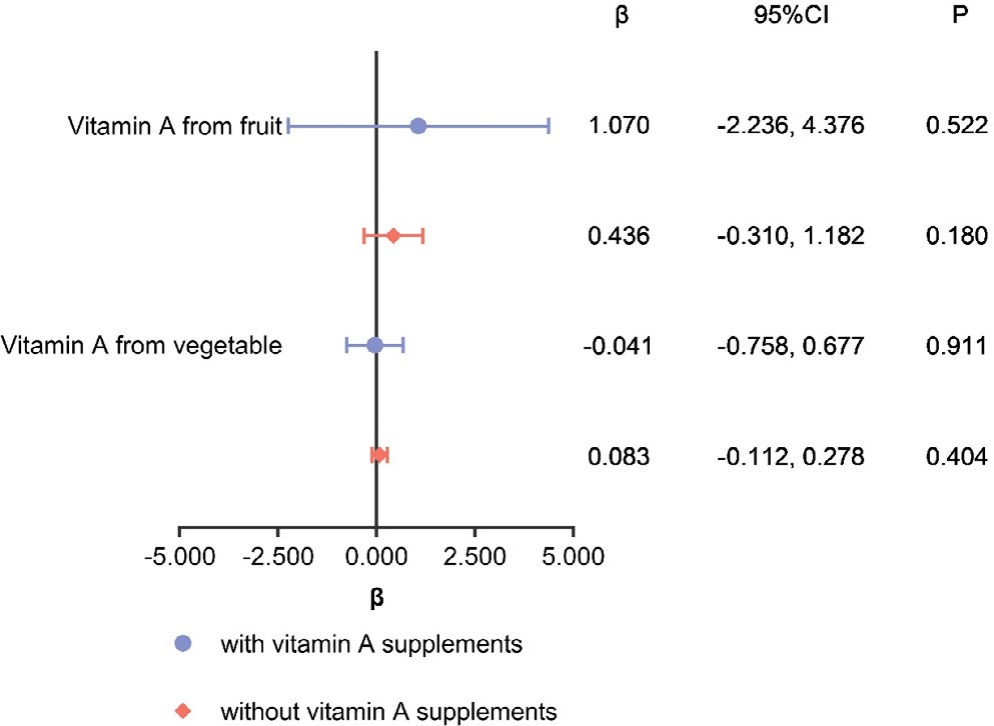
Stratified analysis of association between vitamin A intake from fruit and vegetable (μg RAE/day) and serum vitamin A concentration (μmol/ml)

The median (25th to 75th) dietary vitamin A intake was 349.5 (202.5–591.4) μg RAE/day. Vegetable contributed 24.9% and fruit 4.8% of the dietary vitamin A intake. After adjustment for possible influential factors, no statistically significant association was found of vitamin A from vegetable with serum vitamin A concentration (β = 0.071, 95%CI = −0.118, 0.259, *p* = .463), and vitamin A from fruit with serum vitamin A concentration (β = 0.461, 95%CI = −0.294, 1.215, *p* = .168). In the stratified analysis, these associations remained not statistically significant, whether lactating women used vitamin A supplements or not (Figure 4).

## 4 DISCUSSION

In the present study, we found 64.7% and 85.5% of Chinese lactating women failed to meet the fruit and vegetable consumption recommended by the CNS, and *zuo yuezi* was negatively associated with fruit consumption. Furthermore, to the best of our knowledge, this is the first study to describe the association of fruit and vegetable consumption assessed using two different dietary assessments with vitamin A status among Chinese lactating women. Our results suggest that total fruit and vegetable consumption, and fruit consumption were associated with serum vitamin A concentrations.

Fruits and vegetables have been shown to have many health‐promoting benefits because of their low energy density, high nutrient density, dietary fiber content, and a diverse array of dietary bioactive compounds (Wallace et al., 2020). Although the Chinese traditional diet pattern is plant‐based, lactating women are a particular group. Consistent with previous studies (Ding, Niu, et al., 2020; Ding, Indayati, et al., 2020; Hu et al., 2019; Zhao et al., 2016), the present study found a high proportion of inadequate fruit and vegetable consumption during lactation. One qualitative research study in China has reported that lactating women and their family members identify fruits and vegetables perceived as “warm” and “cold” and that they eat fewer kinds of fruits and vegetables, avoiding those that are thought to be “cold” (Raven et al., 2007). This traditional belief plays a role in the intake of fruits and vegetables and the dietary diversity of lactating women. In the present study, we found lactating women during *zuo yuzi* had lower consumption of total fruit and vegetables, and fruits. We did not observe similar results concerning vegetable consumption and green leafy vegetables and colored vegetable consumption. In traditional Chinese cuisine, the vegetable is an important ingredient, but the fruit is usually consumed as a snack and not a necessary dietary component (Y. C. Li et al., 2017). One study focused on the dietary intake status of Chinese lactating women during the first month postpartum, and reported that the vegetable consumption rate was higher than the fruit consumption rate (Duan et al., 2016). Similarly, our results also found that fruit consumption was more unsatisfactory and needed more attention (25th: 0 g/day). Additionally, the vegetable choices were a concern. The present study found green leafy vegetables and colored vegetables made up small proportions of total vegetable consumption.

The present study also found that lactating women aged ≤30 years, without college or university education, and with lower family monthly per capita income had a lower consumption of vegetables. The positive associations of education and income with vegetable consumption have been researched in several studies, but the associations for age have yielded inconsistent results (Kwon et al., 2020; Li et al., 2020; Li et al., 2017; Miller et al., 2016; Tovar et al., 2019). Because our study population was relatively young, more studies will be needed to investigate this aspect further. Additionally, lactating women who resided in third‐tier or fourth‐tier cities, and those who resided in the north of China had lower consumption of fruit and vegetable. Our findings confirmed the pronounced effects of economic level on fruit and vegetable consumption, and the different dietary habits and preferences between northern and southern Chinese (Li et al., 2017; Qin et al., 2015). In conclusion, our findings indicate the need to increase the intake of fruits and vegetables in lactating women, while making better vegetable choices at the same time. Lactating women during *zuo yuezi*, with low education and with low income, should be the focus of future prevention work.

During pregnancy and throughout the breastfeeding period, vitamin A has an essential role in the healthy development of the fetus and newborn, with lung development and maturation being particularly important (Strobel et al., 2007). Vitamin A cannot be produced by humans and must be provided as part of the diet. Insufficient vitamin A intake by the mother negatively affects breast milk and may be potentially detrimental to the lactating infant (Machado et al., 2019). Vitamin A deficiency is the main global cause of preventable childhood blindness and also increases the risk of mortality from other childhood diseases (Bassey et al., 2020). The Dietary Guidelines for Chinese lactating women recommend that lactating women should consume a diet that is rich in both vitamin A and pro‐vitamin A (Yang et al., 2018). Vitamin A is found in animal‐based foods such as retinyl esters (mainly retinyl palmitate). In fruits and vegetables, it occurs as pro‐vitamin A carotenoids (mainly β‐carotene, α‐carotene, and β‐cryptoxanthin), which can be cleaved and metabolized into retinol after absorption by intestinal cells (Reboul, 2013). Nutrient supplements are also important sources of vitamin A. In the present study, lactating women with vitamin A supplements had higher serum vitamin A concentrations than those without vitamin A supplements.

We used 24HDR data and SFFQ data to explore the associations of fruit and vegetable consumption and serum vitamin A concentrations, respectively. We found strong interrelations between the various food groups assessed by the 24HDR and SFFQ. Because dietary fat can promote the absorption and bioconversion of carotenoids (Tanumihardjo, 2002), and animal products are also essential vitamin A sources, we adjusted these two factors in our multivariate analyses. Similar results were observed in our two analyses. Total fruit and vegetable consumption, and fruit consumption were associated with higher serum vitamin A concentrations, and these associations were more pronounced among lactating women without vitamin A supplements. It has been long recognized that fruit and vegetable consumption is essential to vitamin A status (Alaofè et al., 2017; Maina et al., 2019; Strobel et al., 2007). However, we did not observe the positive association between vegetable consumption and serum vitamin A concentrations in our two analyses. One possible explanation is the difference between fruits and vegetables as sources of pro‐vitamin A. The bioavailability and bioconversion are lower from vegetables and better from orange fruit (Tanumihardjo et al., 2016). Carotenoids in fruits are found mainly in chromoplasts and are more efficiently released by digestion than carotenoids in green vegetables, which are primarily located in chloroplasts (Mercadante, 2019; Tanumihardjo et al., 2016). Another possible explanation was the wide range of vegetables. Not all vegetables are a good source of pro‐vitamin A. Previous studies have reported the association between darkly colored vegetables and vitamin A status (Egbi et al., 2018; Tang et al., 1999). However, one intervention study has reported that there is no effect of African leafy vegetable consumption on vitamin A status in children (van der Hoeven et al., 2016). In the present study, we also observed less stable results. It should be noted that green leafy vegetable and colored vegetable intake were very low in our sample, which might account for these less stable results.

The contribution of fruit and vegetable to dietary vitamin A intake was close to one‐third. However, we did not find any positive associations of vitamin A from fruit and vegetable with serum vitamin A concentrations. We speculated that the possible reason was the limitation of 24HDR, which was not adequately capturing participants' daily nutrients consumption level. Although we found a positive association between fruit and vegetable consumption in the past 24 h and serum vitamin A, it may simply be because lactating women with high fruit and vegetable intake in the past 24 h had a habit of eating fruit and vegetable, and the correlations between 24HDR and SFFQ help demonstrate this to some extent. We speculated that dietary habits with high fruit and vegetable were the key reason for high serum vitamin A. It has been estimated that for healthy, well‐nourished individuals, ~70% of vitamin A present in the body is stored in the liver (Borel & Desmarchelier, 2017; O'Byrne & Blaner, 2013). Good dietary habits may play an important role in maintaining adequate liver storage, which was subsequently reflected in the blood.

Interestingly, although lactating women during *zuo yuezi* had relatively lower total fruit and vegetable consumption, there was no difference in serum vitamin A concentrations between lactating women during *zuo yuezi* and after *zuo yuezi*. One of the possible explanations was the dietary variation between these two groups. In this study, we found lactating women during *zuo yuezi* had higher consumption of eggs, which was a good source of vitamin A. Eggs are considered among the most beneficial foods for maternal and infant health and are the most commonly consumed animal source food during *zuo yuezi*. Another possible explanation was liver storage. Lower dietary vitamin A intake means that liver storage tends to be used (da Silva et al., 2019). Lower dietary intake coupled with depleted liver storage of vitamin A predisposes mothers to vitamin A deficiency (Baytekus et al., 2019). However, we could not determine the liver vitamin A storage in the present study. Modified relative dose–response test is recommended and should provide a qualitative measurement of low or adequate liver vitamin A storage in the future (Tanumihardjo et al., 2016).

Our study had some limitations. First, the sample size of participants during *zuo yuezi* at the time of the survey was relatively small, which may not be enough to reflect the food consumption of lactating women during *zuo yuezi*. Furthermore, we did not differentiate the food consumption between women staying at home and living in professional facilities (e.g., “*yue zi* center”), which may affect women's diet. Second, the study was cross‐sectional in design; thus, it can only determine associations but not causality. The causal relations between fruit and vegetable consumption and vitamin A status need to be adequately examined with longitudinal studies in the future. Third, the study only included serum vitamin A concentrations; we did not assess other blood indicators, for example, β‐carotene, meaning that this was not comprehensive enough. One study has reported that fruit and vegetable consumption is associated with a higher β‐carotene concentration, but not associated with a higher retinol concentration (Mielgo‐Ayuso et al., 2017). Fourth, 24HDR and SFFQ both have their own sources of limitation, and recall bias was unavoidable. 24HDR was not adequately capturing participants' daily food or nutrients consumption level and, consequently, was not appropriate for reporting insufficient intake. We only conducted one‐time 24HDR without distinguishing workdays and weekends. Since this study focused on lactating women, most of them (76.9%) were not working (e.g., on maternity leaves or homemakers). We supposed variation in food consumption on workdays and weekends was not too large. We could not calculate energy intake based on SFFQ; thus, we did not adjust this factor in the models for the association. Fifth, seasonal variation in fruit and vegetable intake should be considered. However, our survey lasted more than a year and covered four seasons; thus, seasonal changes in food availability might be less important. Besides, the survey was performed in ten cities in urban China, and the participants had a relatively higher socioeconomic status and usually had more food choices. Hence, we supposed seasonal variation might be very limited. Finally, the extrapolation of the conclusion is limited. One survey has reported that lactating women in rural China had relatively lower fruit and vegetable intakes than those in urban China (Ding, Niu, et al., 2020; Ding, Indayati, et al., 2020;). Future research should provide more attention to rural areas in China.

## 5 CONCLUSIONS

In conclusion, this cross‐sectional study found that Chinese lactating women in ten cities had inappropriate fruit and vegetable intake. Total fruit and vegetable consumption and fruit consumption assessed using either the 24HDR or SFFQ were positively associated with serum vitamin A concentrations, respectively. Because *zuo yuezi* is accepted by Chinese mothers as a special ritual with food restrictions, vitamin supplements or food fortification are perhaps an effective solution.

## CONFLICT OF INTEREST

The authors declare that they have no conflict of interest.

## AUTHOR CONTRIBUTION

**Chenlu Yang:** Formal analysis (equal); Investigation (equal); Methodology (equal); Writing‐original draft (lead). **Ai Zhao:** Conceptualization (equal); Investigation (equal); Methodology (equal); Writing‐review & editing (equal). **Hanglian Lan:** Investigation (equal); Methodology (equal); Writing‐review & editing (equal). **Jian Zhang:** Formal analysis (equal); Investigation (equal); Methodology (equal). **Zhongxia Ren:** Formal analysis (equal); Investigation (equal); Methodology (equal). **Ignatius Man‐Yau Szeto:** Conceptualization (equal); Writing‐review & editing (equal). **Peiyu Wang:** Conceptualization (equal); Writing‐review & editing (equal). **Yumei Zhang:** Conceptualization (equal); Writing‐review & editing (equal).

## ETHICAL APPROVAL

The study was conducted according to the guidelines laid down in the Declaration of Helsinki, and all procedures involving human subjects were ethically approved by the Medical Ethics Research Board of Peking University (No. IRB00001052‐19045). Those who voluntarily decided to participate signed an informed consent form.

## Supporting information

Table S1
