# Supplementary material for: Fruit and vegetable consumption and serum vitamin A in lactating women: A cross‐sectional survey in urban China
Source: Food Sci Nutr. 2021 Aug 20;9(10):5676–88. doi: 10.1002/fsn3.2532 (PMC8497839; doi:10.1002/fsn3.2532)
Supplement: Supplementary file 1 — Table S1 [file FSN3-9-5676-s001.docx]

**Table S1. Summary of correlations of each food group between the 24HDR and SFFQ assessments.**

| SFFQ assessments | 24HDR assessments | | | | | |
| --- | --- | --- | --- | --- | --- | --- |
|  | Total fruit and vegetable | Fruit | Vegetable | Green leafy vegetable and colored vegetable | Cooking oil | Animal products |
| Total fruit and vegetables | 0.444 * |  |  |  |  |  |
| Fruit |  | 0.499 * |  |  |  |  |
| Vegetables |  |  | 0.362* |  |  |  |
| Green leafy vegetables and colored vegetables |  |  |  | 0.337 * |  |  |
| Cooking oil |  |  |  |  | 0.657 * |  |
| Animal products |  |  |  |  |  | 0.367 * |

* P < 0.01
